# Supplementary material for: Genome and transcriptome evolve separately in recently hybridized Trichosporon fungi
Source: Commun Biol. 2019 Jul 19;2:263. doi: 10.1038/s42003-019-0515-2 (PMC6642101; doi:10.1038/s42003-019-0515-2)
Supplement: Supplementary file 1 — Supplementary Information [file 42003_2019_515_MOESM1_ESM.pdf]

Supplementary Figures

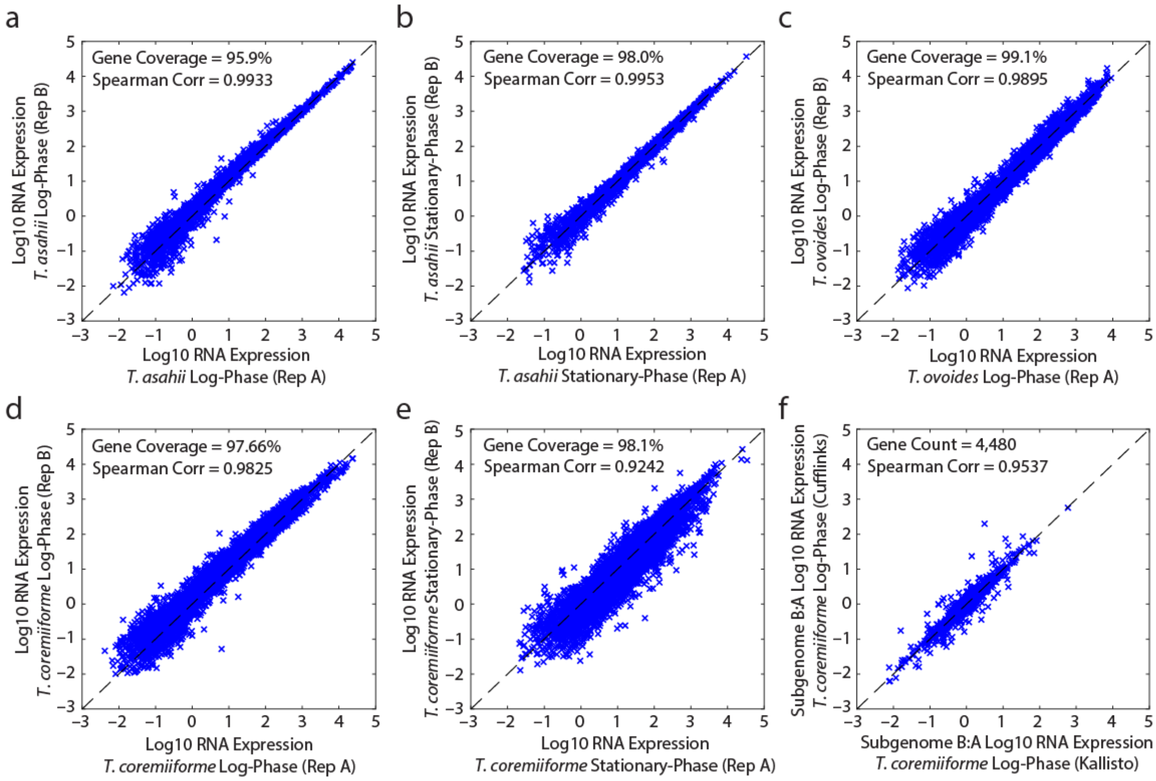

Supplementary Figure 1: Evaluation of RNA-Seq reproducibility and ability to distinguish subgenome-specific transcripts by Cufflinks and Kallisto. Data shown were processed by Kallisto unless indicated otherwise, and the unit of transcript abundance is transcript per million (TPM). Gene coverage represents the fraction of predicted genes whose transcripts were detected in at least one of the replicates. Spearman rank correlation is also indicated. Only genes with nonzero abundances are plotted. **a**) Replicates A and B of *T. asahii* log-phase data. **b**) *T. asahii* stationary-phase data. **c**) *T. ovoides* log-phase data. **d**) *T. coremiiforme* log-phase data. **e**) *T. coremiiforme* stationary-phase data. **f**) Comparison of Cufflinks and Kallisto's performance in distinguishing subgenome-specific homeologs in *T. coremiiforme*. Ratios of subgenome B transcript abundance over subgenome A transcript abundance are shown for 4,480 two-copy homeolog groups with nonzero abundance.

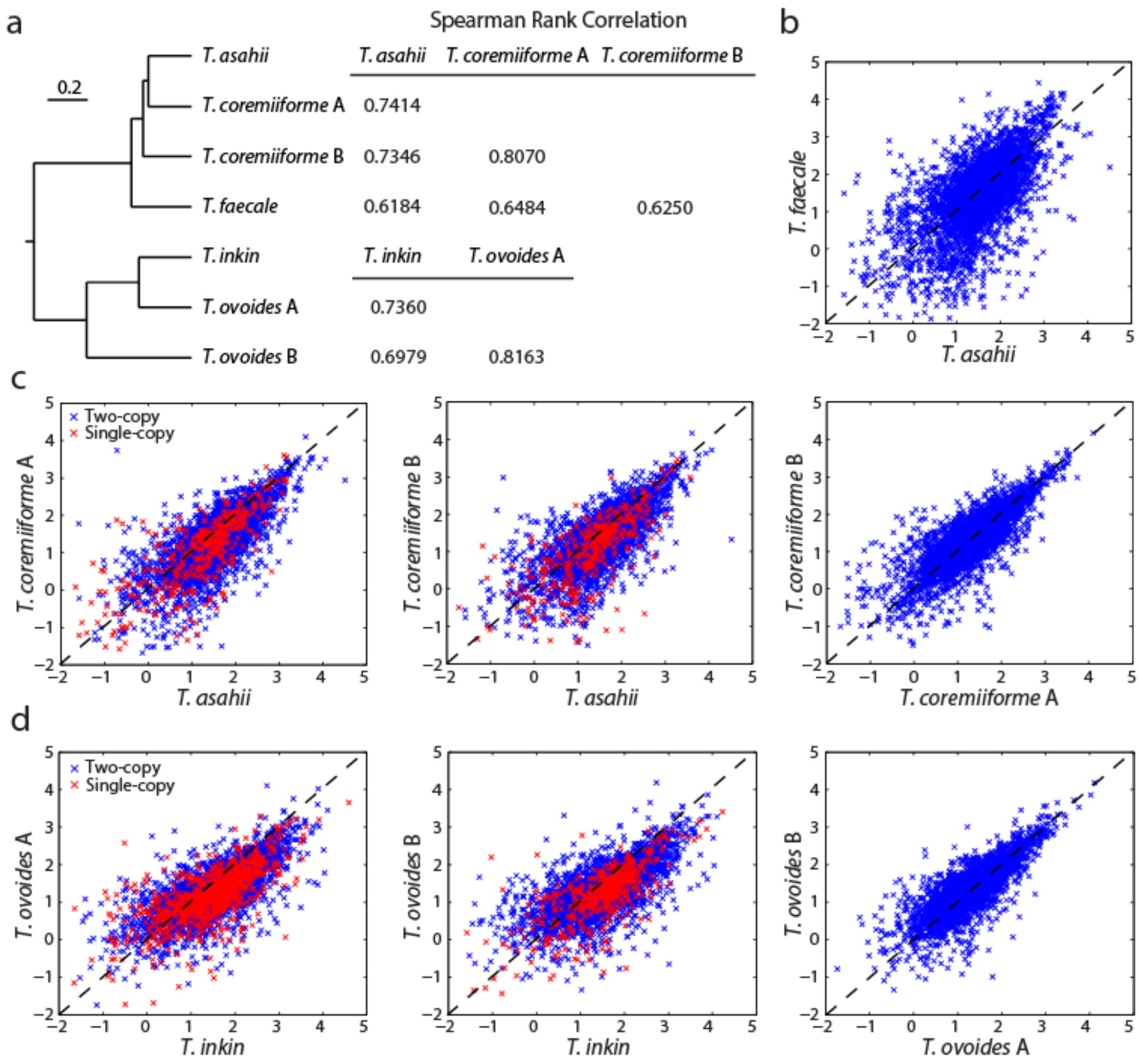

Supplementary Figure 2: Rapid convergences of transcriptional regulation of homeologous genes following genome hybridization. Similar to Figure 1 but displayed using expression levels from stationary-phase growth condition instead. **a**) Phylogenetic relationship between *Trichosporon* species analyzed. Spearman rank correlation coefficients for pairwise comparisons of expression levels between orthologs and homeologs from different *Trichosporon* genome and subgenomes are indicated. Only gene ortholog groups that are present in all species involved (*T. asahii*, *T. faecale*, and *T. coremiiforme* for the top table and *T. inkin* and *T. ovoides* for the bottom table) were included in the calculations. **b**) Scatter plot comparing expression levels in log10 fragments per kilobase of transcript per million mapped reads (FPKM) between *T. asahii* and *T. faecale* orthologs. Dashed lines indicate the $x = y$ diagonal. **c**) Similar scatter plots for the comparisons of expression levels between *T. coremiiforme*'s subgenomes and *T. asahii*. Data points corresponding to two-copy and single-copy homeolog groups in *T. coremiiforme* are distinguished by blue and red markers, respectively. **d**) Similar scatter plots for the comparisons of expression levels between *T. ovoides*'s subgenomes and *T. inkin*.

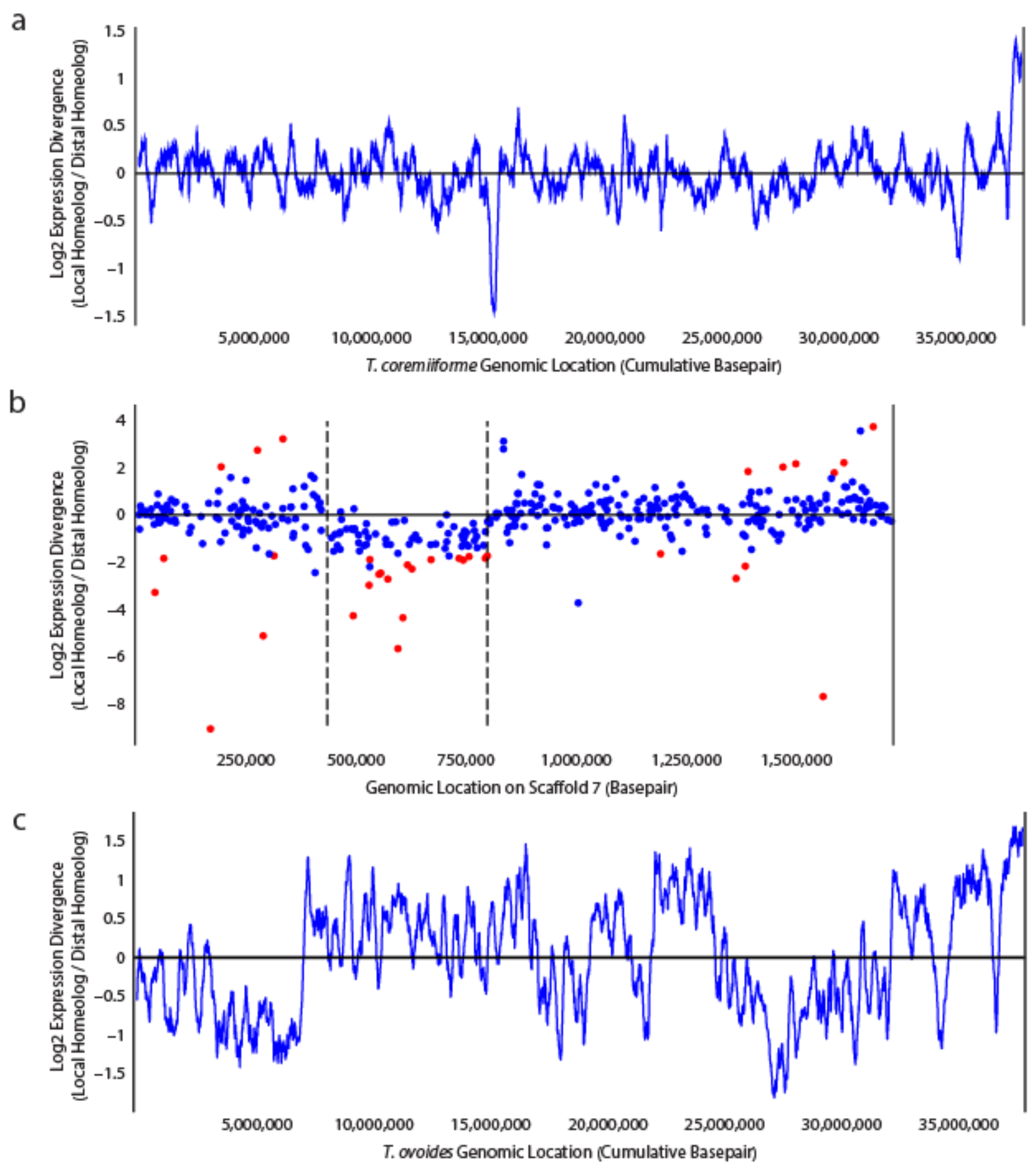

Supplementary Figure 3: **Suppression of transcriptional activity on a 370kb region in *T. coremiiforme*.** **a**) Line plot showing the moving average (window size = 51) of expression divergence ratio, defined as the ratio of transcript expression level of local gene over that of its homeologous counterpart on another subgenome, along the genome of *T. coremiiforme*. Single-copy genes not were not considered. **b**) Scatter plot showing individual gene's expression divergence ratio along scaffold 7 of *T. coremiiforme*. Dashed lines designate the 370kb region where transcriptional activities are distinctively suppressed. This region corresponds to the clear dip in expression divergence ratio around basepair 15,000,000 observed in **a**). **c**) Similar plot for *T. ovoides*. Here, the log expression divergence ratios do not center around zero because of transcriptional activity bias toward subgenome B.

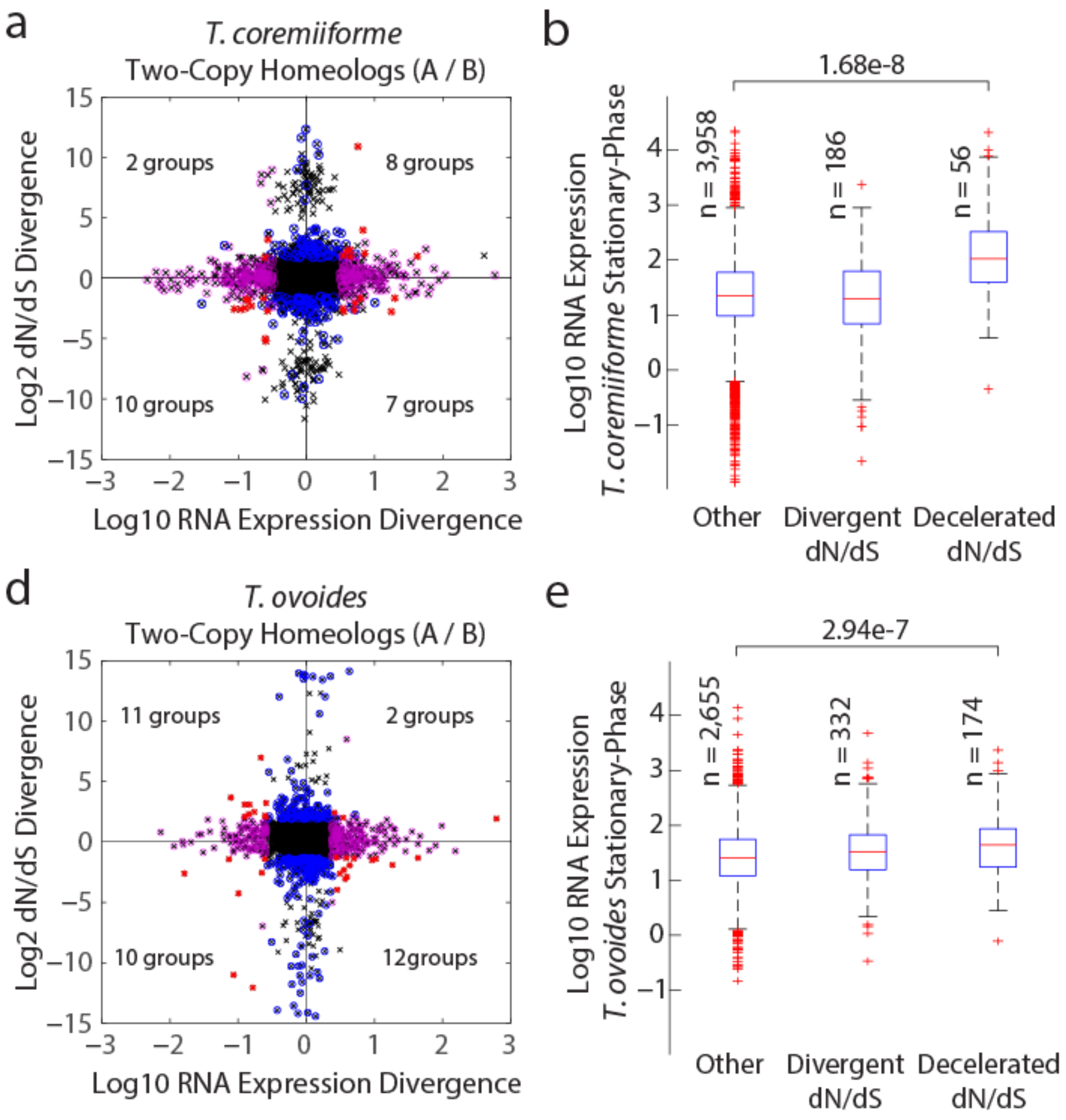

Supplementary Figure 4: Lack of concerted evolution at sequence and expression level among two-copy homeolog groups. Similar to Figure 2 but displayed using expression levels from stationary-phase growth condition. **a**) Scatter plot comparing divergence in evolutionary rate (dN/dS ratio) and divergence in expression level for two-copy homeolog pairs in *T. coremiiforme*. Divergences were calculated as the ratios of subgenome A homeolog's over subgenome B homeolog's. Black x markers display the data for all two-copy homeolog pairs. Blue and magenta circles indicate homeolog pairs with significant divergence in only evolutionary rate or only expression level, respectively (adjusted p-value ≤ 0.01 and fold-difference ≥ 3, see Methods). Red asterisks indicate homeolog groups with significant divergence in both evolutionary rate and expression level and the number of these homeolog groups are indicated in each quadrant. Expression levels from log-phase growth condition are shown. **b**) Box plots

showing log-phase expression level of two-copy homeolog pairs in *T. coremiiforme* with divergent evolutionary rates or decelerated evolutionary rates compared to *T. asahii*'s orthologs (see Methods). Mann-Whitney U test p-value for the comparison between homeolog pairs with decelerated evolutionary rates and those without is indicated at the top. The numbers of homeolog pairs belonging to each class are indicated next to the corresponding box plot. Blue boxes designate the 25th-75th percentile ranges. Red bars indicate the medians. Black whiskers designate the approximated 0.35^{th}-99.65^{th} percentile ranges. Red cross markers indicate individual data points lying outside the 0.35^{th}-99.65^{th} percentile ranges. **c-d**) Similar plots for *T. ovoides*-*T. inkin* comparison.

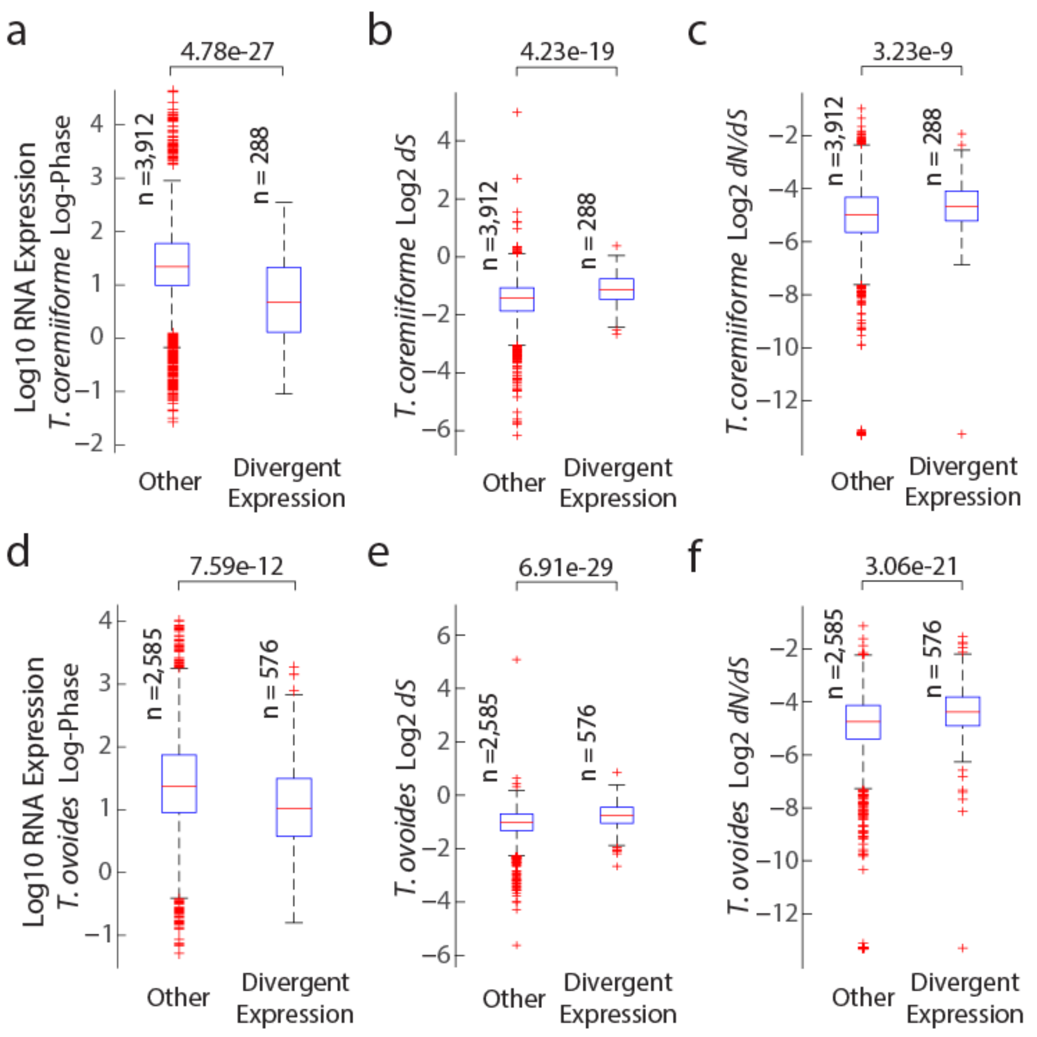

Supplementary Figure 5: Divergent expression of homeolog pairs with low expression and low sequence conservation levels. Expression levels from log-phase growth condition are shown. **a**) Box plots comparing the expression levels between two-copy homeolog pairs in *T. coremiiforme* with significantly divergent expression (between subgenome A and B homeologs) and those without. Blue boxes designate the 25th-75th percentile ranges. Red bars indicate the medians. Black whiskers designate the approximated 0.35^{th}-99.65^{th} percentile ranges. Red cross markers indicate individual data points lying outside the 0.35^{th}-99.65^{th} percentile ranges. **b**) Box plots comparing the mutation rate, as represented by synonymous substitution rate (*dS*), between two-copy homeolog pairs in *T. coremiiforme* with significantly divergent expression and those without. **c**) Box plots comparing the evolutionary rate, as represented by the ratio of nonsynonymous substitution rate to synonymous substitution rate (*dN*/*dS*), between two-copy homeolog pairs in *T. coremiiforme* with significantly divergent expression and those without. **d-f**) Similar box plots for comparisons among *T. ovoides*'s homeolog pairs.

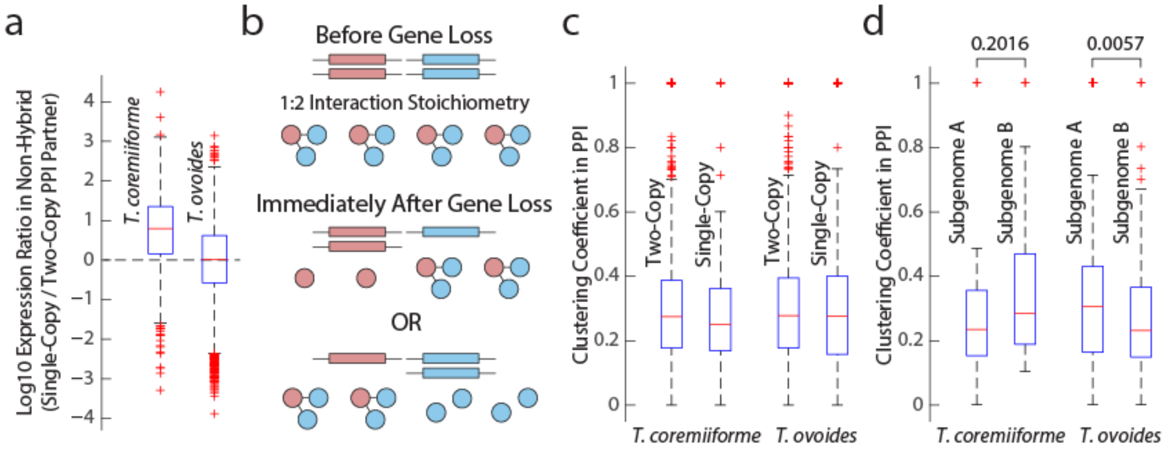

Supplementary Figure 6: Preferential losses of protein-protein interaction partners with higher expression level in *T. coremiiforme* and retentions of genes in highly connected regions of protein-protein interaction network in *T. ovoides*. a) Box plots showing the ratio of expression level (in non-hybrid reference) between the single-copy interaction partner and the two-copy interaction partner for all inferred protein-protein interactions that involve a single-copy gene and a two-copy homeolog pair. Expression levels in *T. asahii* is shown for *T. coremiiforme* and expression levels in *T. inkin* is shown for *T. ovoides*. Expression levels in hybrid species are not used because the evolutionary processes following gene losses may have altered the expression level of these homeologs. Blue boxes designate the 25th-75th percentile ranges. Red bars indicate the medians. Black whiskers designate the approximated 0.35^{th}-99.65^{th} percentile ranges. Red cross markers indicate individual data points lying outside the 0.35^{th}-99.65^{th} percentile ranges. **b**) Schematic showing the immediate impact of gene loss on the amount of unbounded proteins when the lost gene has either higher or lower expression. In general, loss of interaction partner with higher expression is expected to result in less amount of unbounded protein products (the middle illustration) than the loss of interaction partner with lower expression (the bottom illustration). **c**) Box plots comparing clustering coefficients (based on the protein-protein interaction network of *S. cerevisiae*) between homeolog groups that retain both gene copies and those that have lost one gene. Mann-Whitney U test p-values are indicated. **d**) Similar to box plots shown in **c**) but comparing clustering coefficients between single-copy genes located on subgenome A and subgenome B instead

Supplementary Tables

Transcription Factor	Copy number in *T. coremiiforme*	Copy number in *T. ovoides*
ARG81	Two-copy	Two-copy
ARO80	Two-copy	Two-copy
ASG1	Two-copy	A
GAL4	Two-copy	Two-copy
HAP3	Two-copy	Two-copy
KAR4	Two-copy	B
MBP1	Two-copy	Two-copy
NHP6A or NHP6B	Single-copy (unassigned)	A
PIP2	Two-copy	Two-copy
PPR1	Two-copy	Two-copy
PUT3	Single-copy (unassigned)	Two-copy
RDS2	Two-copy	A
SKN7	Two-copy	Two-copy
SPT15	Two-copy	A
STB5	Two-copy	A
TEA1, CHA4	Single-copy (unassigned), Not found	A, Two-copy
UGA3	Two-copy	A

Supplementary Table 1: Gene loss pattern for transcription factors in *Trichosporon* hybrids. NHP6A and NHP6B were mapped to the same ortholog group in *Trichosporon* species. Some orthologs in *T. coremiiforme* are located in regions that cannot be assigned to specific subgenomes and are indicated as unassigned. TEA1 and CHA4 were together mapped to two ortholog groups in *Trichosporon* species but there was not enough data to determine which group is TEA1 and which is CHA4.

	T. coremiiforme				*T. ovoides*			
TF	Sub A	Sub B	Shared	P-value	Sub A	Sub B	Shared	P-value
ARG81	549 (85)	546 (82)	249 (37)	< 1e-11	474 (66)	541 (63)	115 (15)	< 1e-11
ARO80	854 (155)	866 (145)	373 (51)	< 1e-11	651 (131)	826 (155)	225 (23)	< 1e-11
CHA4	1525 (251)	1529 (262)	801 (109)	< 1e-11	962 (212)	996 (196)	301 (35)	1.48e-06
GAL4	739 (99)	741 (120)	231 (25)	< 1e-11	613 (98)	590 (71)	118 (5)	1.05e-03
HAP3	632 (89)	662 (97)	344 (53)	< 1e-11	518 (68)	573 (90)	191 (19)	< 1e-11
MBP1	325 (59)	341 (63)	156 (30)	< 1e-11	358 (52)	310 (64)	91 (10)	< 1e-11
NHP6A	1029 (270)	1045 (245)	560 (113)	< 1e-11	720 (180)	875 (207)	318 (60)	< 1e-11
NHP6B	942 (235)	947 (221)	493 (98)	< 1e-11	654 (147)	809 (167)	266 (37)	< 1e-11
PUT3	360 (48)	332 (42)	117 (13)	< 1e-11	498 (91)	353 (66)	75 (9)	9.89e-07
RDS2	1109 (44)	1117 (37)	523 (8)	< 1e-11	432 (48)	424 (58)	69 (7)	2.20e-04
SPT15	1078 (300)	1135 (306)	598 (161)	< 1e-11	777 (224)	926 (249)	331 (78)	< 1e-11
STB5	802 (132)	825 (140)	388 (66)	< 1e-11	629 (120)	623 (120)	211 (36)	< 1e-11
TEA1	726 (100)	704 (117)	296 (40)	< 1e-11	654 (105)	605 (109)	150 (11)	1.36e-08
UGA3	892 (106)	874 (110)	381 (42)	< 1e-11	822 (179)	676 (134)	221 (36)	< 1e-11

Supplementary Table 2: Enrichment of shared transcription factor binding sites in 1,000-bp or 300-bp upstream regions of the start codon of 4,686 two-copy homeolog pairs in *T. coremiiforme* and 3,903 two-copy homeologs pairs in *T. ovoides*. Sub A and Sub B indicate subgenomes A and B, respectively. The numbers of identified transcription factor binding sites are indicated. The results for 1,000-bp upsteam regions are indicated without parentheses. The results for 300-bp upstream regions are indicated inside parentheses. Hypergeometric test p-values for the analyses of 1-kb upstream regions are reported here. P-values smaller than 1e-11 are reported as <1e-11.

Group 1: 288 *T. coremiiforme* two-copy homeolog pairs with at least 3-fold divergent log-phase RNA expression					
Accession ID	Description	Term Type	Local Annotated	Global Annotated	Adjusted P-value
GO:0055085	transmembrane transport	biological_process	30	318	1.70e-4
GO:0016021	integral component of membrane	cellular_component	30	346	9.39e-4
PF13193	AMP-binding enzyme C-terminal domain	pfam id	5	19	0.0435
Group 2: 576 *T. ovoides* two-copy homeolog pairs with at least 3-fold divergent log-phase RNA expression					
Accession ID	Description	Term Type	Local Annotated	Global Annotated	Adjusted P-value
GO:0016021	integral component of membrane	cellular_component	48	346	1.62e-3
GO:0055085	transmembrane transport	biological_process	45	318	1.83e-3
GO:0055114	oxidation-reduction process	biological_process	44	343	0.0242
Group 3: 326 *T. ovoides* two-copy homeolog pairs with at least 3-fold divergent stationary-phase RNA expression					
Accession ID	Description	Term Type	Local Annotated	Global Annotated	Adjusted P-value
GO:0055085	transmembrane transport	biological_process	37	318	8.97e-7
GO:0016021	integral component of membrane	cellular_component	37	346	8.45e-6
Group 3: 54 Two-copy homeolog pairs with at least 3-fold divergent RNA expression in at least 3 out of 4 conditions					
Accession ID	Description	Term Type	Local Annotated	Global Annotated	Adjusted P-value
GO:0055085	transmembrane transport	biological_process	10	318	1.28e-3
GO:0016021	integral component of membrane	cellular_component	9	346	0.0131

Supplementary Table 3: Significant functional enrichments for homeolog pairs with divergent expression levels. GO and Pfam terms with fewer than 10 annotated genes (too specific) or more than 500 genes (too broad) were removed from consideration. Hypergeometric distribution p-values were divided by the number of GO and Pfam terms according to Bonferroni correction method and reported here as adjusted p-values.

Entry ID	Gene Name (*T. asahii*)	Annotations
1	3801_contig2_gene960	purine permease, nucleoside transporter
2	3801_contig5_gene4899	MFS general substrate transporter
3	3801_contig14_gene1312	MFS sugar transporter
4	3801_contig19_gene1787	vacuolar amino acid permease
5	3801_contig15_gene28	carboxylic acid transporter
6	3801_contig4_gene2325	related to monocarboxylase permease
7	3801_contig0_gene5636	unknown function, with MFS domain
8	3801_contig0_gene6086	unknown function, with MFS domain
9	3801_contig2_gene723	MFS alpha-glucosine transporter
10	3801_contig15_gene70	related to Na+/H+ exchanger

Supplementary Table 4: Detailed annotations for 10 two-copy homeolog pairs with at least 3-fold divergent expression in both hybrids that were also annotated with GO:005585 Transmembrane transport. Annotations were inferred from BLASTP search against NCBI's non-redundant (nr) protein database.
