# Supplementary material for: Genome and transcriptome evolve separately in recently hybridized Trichosporon fungi
Source: Commun Biol. 2019 Jul 19;2:263. doi: 10.1038/s42003-019-0515-2 (PMC6642101; doi:10.1038/s42003-019-0515-2)
Supplement: Supplementary file 2 — Description of Additional Supplementary Files [file 42003_2019_515_MOESM2_ESM.docx]

Description of supplementary data

**Supplementary Data 1**

Table of evolutionary rates (dN, dS, and dN/dS) and transcript expression levels in fragment per kilobase of exon per million reads mapped (FPKM) for both log and stationary growth phases for *T. asahii* and *T. coremiiforme* genes. Only homeolog groups that remain as two-copy in *T. coremiiforme* are included. These data were used to plot Figure 2A-B.

**Supplementary Data 2**

Table of evolutionary rates (dN, dS, and dN/dS) and transcript expression levels in fragment per kilobase of exon per million reads mapped (FPKM) for both log and stationary growth phases for *T. inkin* and *T. ovoides* genes. Only homeolog groups that remain as two-copy in *T. ovoides* are included. These data were used to plot Figure 2C-D.

**Supplementary Data 3**

Table of protein-protein interaction partners’ transcript expression levels in fragment per kilobase of exon per million reads mapped (FPKM) for both log and stationary growth phases for *T. asahii* and *T. coremiiforme*. These data were used to plot Figure 3B-C.

**Supplementary Data 4**

Table of protein-protein interaction partners’ transcript expression levels in fragment per kilobase of exon per million reads mapped (FPKM) for both log and stationary growth phases for *T. inkin* and *T. ovoides*. These data were used to plot Figure 3D-E.

**Supplementary Data 5**

Table of clustering coefficients for each gene in *T. coremiiforme* and *T. ovoides* in the context of *S. cerevisiae* protein-protein interaction network. Gene copy number and the subgenomic location for each single-copy gene are indicated.
